# Influence of HIV co-infection on clinical presentation and disease outcome in hospitalized adults with tuberculous meningitis in Brazil: a nationwide observational study

**DOI:** 10.3389/fpubh.2025.1600104

**Published:** 2025-06-16

**Authors:** Lucas G. Urmenyi, Caian L. Vinhaes, Klauss Villalva-Serra, Mariana Araujo-Pereira, Bruno B. Andrade

**Affiliations:** ^1^Curso de Medicina, Universidade Salvador, Salvador, Brazil; ^2^Multinational Organization Network Sponsoring Translational and Epidemiological Research (MONSTER) Initiative, Salvador, Brazil; ^3^Instituto de Pesquisa Clínica e Translacional, Faculdade Zarns, Clariens Educação, Salvador, Brazil; ^4^Laboratório de Pesquisa Clínica e Translacional, Instituto Gonçalo Moniz, Fundação Oswaldo Cruz, Salvador, Brazil; ^5^Departamento de Infectologia, Hospital das Clínicas, Faculdade de Medicina da Universidade de São Paulo, São Paulo, Brazil

**Keywords:** tuberculous meningitis, HIV, clinical presentation, risk factors, Brazil

## Abstract

**Introduction:**

Tuberculous meningitis (TBM) is a severe form of central nervous system infection caused by *Mycobacterium tuberculosis* (Mtb) that is often associated with significant morbidity and mortality, particularly in people living with HIV (PLWH). This study investigated differences in clinical and laboratory profiles of TBM cases in Brazil associated with HIV status, and identified factors associated with in-hospital mortality.

**Methods:**

We conducted a retrospective analysis of 1,819 hospitalized adult TBM patients reported in the Brazilian Notifiable Diseases Information System (SINAN) meningitis database from 2007 to 2021. Confirmed cases in hospitalized individuals aged >18 years with known HIV status were included; pregnant patients were excluded. Clinical and laboratory features were compared by HIV status and clinical outcomes. Classification and regression tree analysis was used to identify outcome-based cut-off values for selected continuous variables. Associations with in-hospital mortality were assessed using backward stepwise binomial logistic regressions.

**Results:**

The majority (57%) of TBM cases comprised of PLWH, who exhibited lower frequencies of vomiting, nuchal rigidity, signs of meningeal inflammation, and coma, along with lower leukocyte counts in cerebrospinal fluid (CSF) compared to HIV-negative patients. PLWH also displayed lower mortality rates (17.3% vs. 23.2%, *p* = 0.002). Features independently associated with mortality included seizures (aOR: 2.15, 95%CI: 1.39–3.33, *p* < 0.001), nuchal rigidity (aOR: 1.57, 95%CI: 1.1–2.23, *p* = 0.014), age > 64 years old (aOR: 2.11, 95%CI: 1.08–4.13, *p* = 0.03), CSF protein concentration ≥441 mg/dL (aOR: 2:08, 95%CI: 1.39–3.09, *p* < 0.001) and CSF glucose concentration ≥ 22 mg/dL (aOR: 0.54, 95%CI: 0.38–0.76, *p* < 0.001), but not HIV (OR: 0.73, [95%IC: 0.52–1.01], *p* = 0.06).

**Conclusion:**

Our findings suggest that despite greater prevalence in PLWH, these patients present fewer clinical signs and symptoms and lower mortality rates. Additionally, HIV was not an independent predictor of mortality in this study population.

## 1 Introduction

Tuberculous meningitis (TBM) is a severe form of extrapulmonary tuberculosis (EPTB) with high morbidity and mortality (1), particularly in regions with a high tuberculosis (TB) burden. Despite healthcare advancements, TBM diagnosis, treatment, and disease outcomes persist as dilemmas, especially in the context of the human immunodeficiency virus (HIV) coinfection.

HIV co-infection increases the risk of both pulmonary and extrapulmonary TB (2) and complicates TBM diagnosis due to atypical presentations (3), raising the likelihood of missed or delayed diagnoses. Moreover, the influence of HIV on TBM outcomes is controversial, with conflicting findings in the literature. While several studies have linked HIV co-infection to increased TBM mortality rates (4, 5), others have not demonstrated a significant impact on in-hospital fatality (6–8). Previous investigations have also proposed TBM clinical and laboratory presentations are similar between HIV populations (5, 9–11). However, many of these studies were limited by small sample sizes and restricted to data from one or two reference centers (5, 9, 10). Given the mixed evidence and the limitations of existing studies, further research with larger populations is crucial to better understand the true impact of HIV on TBM clinical presentation and outcomes, ultimately improving patient management.

Brazil ranks among the countries with the highest burden of TB and TB/HIV co-infection (12). TBM represents 6% of all EPTB cases and is the third most common neurological opportunistic infection in the country (13, 14). Despite this, comprehensive nationwide studies on the clinical and epidemiological characteristics of TBM in Brazil are scarce. In this study, we leveraged a large dataset from the Brazilian Information System for Notifiable Diseases (SINAN), covering TBM cases from 2007 to 2021, to characterize the epidemiologic and clinical aspects of hospitalized TBM patients in Brazil. We also sought to identify differences in clinical and laboratory features based on HIV status and determine the factors associated with in-hospital mortality to guide targeted interventions and improve clinical management.

## 2 Methods

### 2.1 Overall study design

This was a retrospective observational study with analysis of data reported in SINAN-meningitis database, from 2007 to 2021. We described and analyzed clinical aspects, laboratory results, sociodemographic characteristics, and outcomes among Brazilians diagnosed with TBM according to HIV status.

### 2.2 SINAN, Brazilian Ministry of Health

SINAN is a system implemented, supported, and maintained by the Brazilian Ministry of Health that registers notifications and investigations of diseases that classify as being of compulsory notification under Brazilian law, which includes various forms of meningitis (e.g., meningococcal, tuberculous, aseptic, pneumococcal, by Haemophilus, among others) (15). In the setting of a suspected or confirmed case, the Brazilian Ministry of Health (BMoH) requires compulsory notification into the system by healthcare professionals. Upon each diagnosis, a standardized form specific to meningitis is filled and supplied into the SINAN system. This form identifies the etiology and collects general variables common to all forms of meningitis including clinical symptoms (trauma, headache, fever, vomiting, seizures, nuchal rigidity, Kernig’s/Brudzinski’s sign, coma, petechial/hemorrhagic suffusions, cerebrospinal fluid appearance), CSF laboratory data (neutrophil and lymphocyte ratio, leukocyte count, and protein and glucose concentration), and sociodemographic data (age, sex, education, self-reported race), among other data. The form is publicly available and can be accessed via the website http://portalsinan.saude.gov.br/meningite. To achieve neutrophil and lymphocyte counts, we divided their respective ratios and multiplied it by the total CSF leukocyte count.

As previously stated, TBM is classified as a disease requiring compulsory notification that can be reported based on initial suspicion. Following investigation, the diagnosis is classified as either confirmed or discarded according to case definitions provided by the Health Surveillance Secretariat and Epidemiological Surveillance Department’s Infectious Diseases guide (16), also maintained by BMoH. Confirmed case definitions go as follows: (i) Every suspected case of meningitis (adults with fever, intense headache, projectile vomiting, nuchal rigidity, signs of meningeal irritation) whose diagnosis is confirmed by the following laboratory tests: smear, culture, or clinical course/evolution; (ii) Every suspected case of meningitis with history of epidemiological linkage to a confirmed case of pulmonary TB. Conversely, discarded case definitions include: (i) every suspected case with confirmed diagnosis for another disease.

Between 2007 and 2021, a total of 406,692 meningitis cases were notified and registered in the SINAN-meningitis database. For this study, the inclusion criteria for data collection required hospitalization and confirmed diagnosis (aforementioned) of TBM, ≥18 years of age, and known HIV status. Cases with pregnancy and those aged <18 years were excluded due to special clinical considerations associated with these subgroups. Pregnant cases were excluded considering the unique physiological and immune changes that take place during pregnancy - which may influence TBM clinical presentation, outcome and thus clinical decision-making. Additionally, cases with unconfirmed diagnosis or unspecified outcome were excluded from the analysis. Furthermore, to account for the possible influence of traumatic taps on CSF leukocyte counts, an arbitrary cut-off of CSF erythrocyte counts > 1,000 cells/mm3 was adopted, excluding those who exceeded this threshold (*n* = 236) or who had missing data (*n* = 1,324) in this field. The application of these criteria resulted in a population of 1,819 cases. Further details on exclusion criteria are provided in Supplementary Figure S1.

### 2.3 Study definitions

In this study, we compared cases whose outcomes were classified as deceased (indicating death by meningitis) or survived (defined as hospital discharge). Outcomes classified as either “death by other causes” or “ignored” were excluded given that the SINAN-meningitis database does not include the exact cause of death. As a result, it is not possible to determine whether these deaths were due to non-biological causes or pre-existing conditions unrelated to TBM. In reference to population, patients self-declared as black, yellow, brown, or indigenous were classified as non-white. Educational level was classified as “literate” or “illiterate.” HIV status was reported according to Brazilian Ministry of Health guidelines (17).

### 2.4 Data analysis

Quantitative variables were described using median and interquartile range (IQR), whereas qualitative variables were presented as percentages (%). Categorical variables were compared using Pearson’s chi-square (𝜒^2^) test. Quantitative variables with two categories were compared using Mann Whitney *U* test. A binomial logistic regression model with a backward stepwise method was employed to evaluate independent associations between sociodemographic, clinical, and laboratory variables with death by TBM. Starting with all candidate variables in the model, this method sequentially removes the least significant predictors (i.e., variables) based on the Akaike information criterion, to arrive at a final, best-fitting model that retains only the most relevant predictors of outcome. Results were presented as adjusted Odds Ratio (OR), with a 95% confidence interval, controlling for multiple confounders. Crude ORs are provided in Supplementary Tables S8–S10. A *p*-value < 0.05 was considered significant in this study. Statistical analysis was performed using R (version 4.4.0).

In order to identify data-driven cut-off values for continuous variables (e.g., age, CSF glucose and protein levels, as well as CSF leukocyte, neutrophil and lymphocyte counts), classification tree analysis were employed using the rpart package in R and visualized with the rpart.plot package. This method identifies thresholds that best separate categorical outcome groups (e.g., hospital discharge *vs.* death by TBM), which allows one to categorize continuous variables into intervals based on these outcome-driven splits (i.e., thresholds). In this way, selected categorized variables described below were then included in the logistic regression models to estimate their association with outcome, using one category as reference.

The following variables were included in the model as potential confounders: sex, race, HIV status, previous TB, headache, fever, vomiting, seizures, nuchal rigidity, Kernig’s/Brudzinski’s sign, CSF neutrophil and lymphocyte counts, CSF glucose (≥22 vs. <22 mg/dL), age (<39, 40–64, >64 years), CSF leukocyte count (<36, 36–64, >64 cells/mm^3^), and CSF protein (≥441 vs. < 441 mg/dL).

Regarding missing data, multivariate analyses were conducted using a complete-cases approach, which includes only individuals with no missing data for the variables included in each model (*n* = 921 for the logistic regression; *n* = 1,218 for the classification tree analysis). For descriptive and univariate comparisons, the compare groups function in R was employed, which handles missing input by reporting the number of non-missing observations for each variable.

### 2.5 Ethical aspects

All data in this study was provided by SINAN, a platform available by the BMoH. This study was conducted following the Resolution No 466/12, which regulates research involving humans in Brazil. All the data were anonymized and publicly available, and the study did not require consent to participate or ethical approval.

## 3 Results

### 3.1 Overall characteristics of the study population

**Figure 1:**
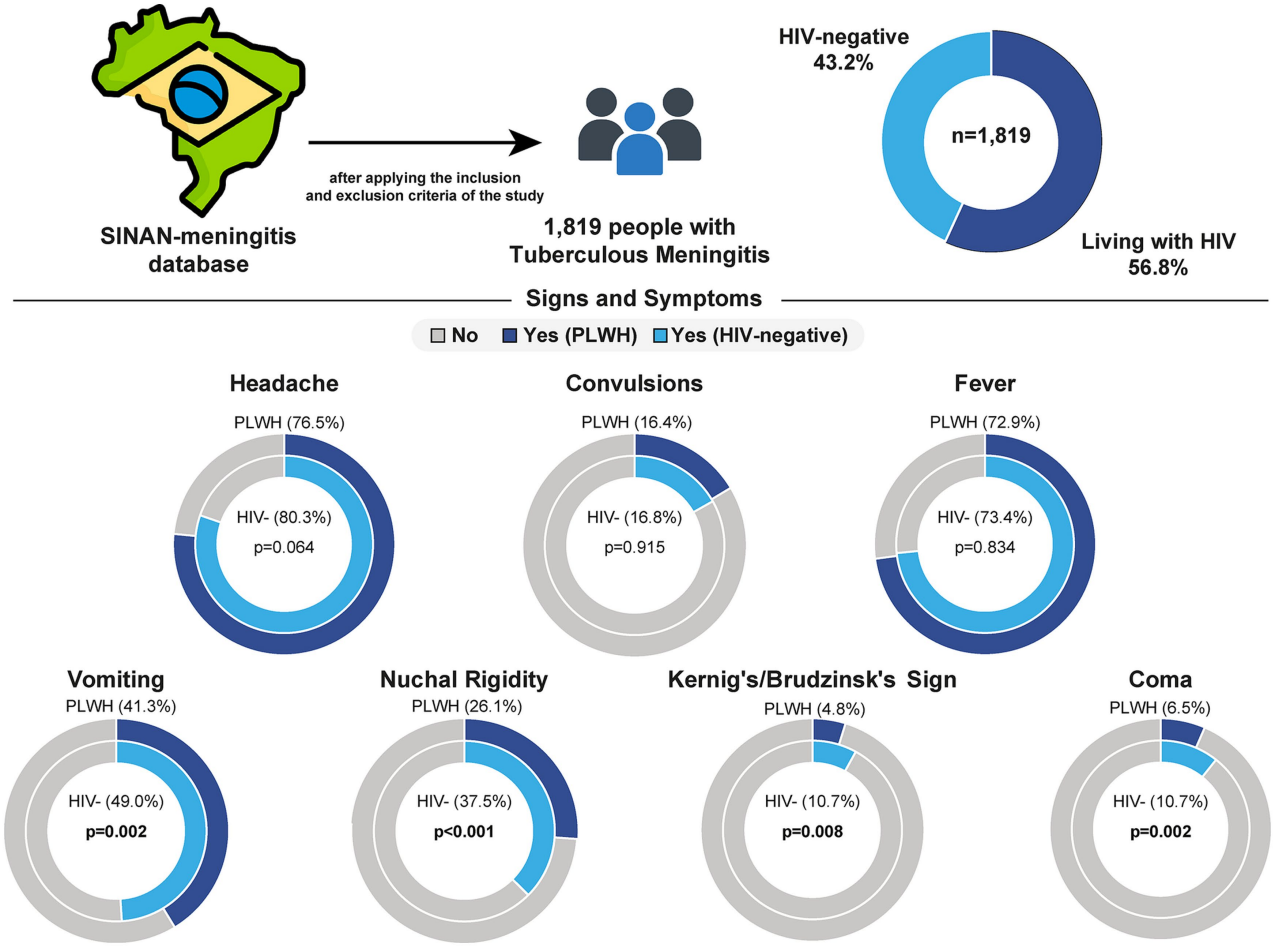
Frequency of clinical signs and symptoms of tuberculous meningitis by HIV status in Brazil. Data from 1,819 individuals with tuberculous meningitis were extracted from the SINAN-meningitis database after applying inclusion and exclusion criteria. The sample included 56.8% people living with HIV (PLWH) and 43.2% HIV-negative individuals. The frequency of seven clinical signs and symptoms was compared between groups. PLWH were less likely to present with vomiting, nuchal rigidity, Kernig’s/Brudzinski’s sign and coma when compared to HIV-negative individuals. No significant differences were observed for headache, convulsions, or fever. HIV, human immunodeficiency virus; PLWH, people living with HIV.

A total of 5,084 patients were registered with TBM in SINAN between 2007 and 2021. For this study, 3,265 patients were removed due to being non-hospitalized, younger than 18 years, pregnant, with unknown HIV status or outcome, or with CSF red blood cell counts > 1,000 cells mm-3 (further information portrayed in Supplementary Figure S1). Among the remaining 1,819 patients included in this study, the prevalence of HIV co-infection was 56.8% (*n* = 1,034). When compared to HIV-negative individuals, PLWH were slightly younger [38 (IQR: 32–45) vs. 40 (IQR: 29–54), *p* = 0.010]. Both groups (HIV− and HIV+) encompassed mostly male (63.9 and 67.9%), and literate (96.6 and 95.8%), respectively, without significant differences between groups. Regarding clinical presentation, previous TB diagnosis was most common within PLWH (58.2% vs. 36.5%, *p* < 0.001). In contrast, signs and symptoms such as vomiting (HIV−: 49.0%; HIV+: 41.3%, *p* = 0.002), nuchal rigidity (HIV−: 37.5%; HIV+: 26.1%, *p* < 0.001), Kernig’s/Brudzinski’s sign (HIV−: 8.03%; HIV+: 4.78%, *p* = 0.008) and coma (HIV−: 10.7%; HIV+: 6.50%, *p* = 0.002) were most frequently observed in the HIV- group (Figure 1). History of trauma or acute renal failure, as well as the presence of headache, fever, seizures, and petechiae/hemorrhagic suffusions, did not differ between groups (Table 1).

**Table 1:** Characteristics of general population and comparison between HIV-positive and HIV-negative adults with TBM.

Characteristics	HIV negative (*N* = 785)	HIV positive (*N* = 1,034)	*p* value
Age, median (IQR)	40.0 (29.0–54.0)	38.0 (32.0–45.0)	**0.01**
Sex (Male), *n* (%)	502 (63.9)	696 (67.3)	0.148
Race (Non-white), *n* (%)	339 (49.7)	488 (51.6)	0.472
Education (literate), *n* (%)	394 (96.6)	590 (95.8)	0.636
ARF, *n* (%)	18 (2.39)	14 (1.45)	0.21
Previous TB, *n* (%)	269 (36.5)	555 (58.2)	**<0.001**
Past Trauma, *n* (%)	19 (2.51)	16 (1.64)	0.273
Headache, *n* (%)	601 (80.3)	753 (76.5)	0.064
Fever, *n* (%)	558 (73.4)	722 (72.9)	0.834
Vomiting, *n* (%)	364 (49.0)	404 (41.3)	**0.002**
Seizures, *n* (%)	125 (16.8)	160 (16.4)	0.915
Nuchal Rigidity, *n* (%)	279 (37.5)	253 (26.1)	**<0.001**
Kernig’s/Brudzinski’s sign, *n* (%)	58 (8.03)	46 (4.78)	**0.008**
Coma, *n* (%)	80 (10.7)	64 (6.50)	**0.002**
Petechiae, *n* (%)	6 (0.80)	6 (0.61)	0.852
Symptom Onset, median (IQR)	12.0 (6.00–27.0)	13.0 (4.00–32.0)	0.768
Death by Meningitis, *n* (%)	182 (23.2)	179 (17.3)	**0.002**
CSF analysis			
Appearance (clear), *n* (%)	406 (56.5)	612 (63.7)	0.001
Leukocyte (cells/mm3), median (IQR)	140 (45.0–334)	93.0 (28.0–245)	**<0.001**
Neutrophil (cells/mm3), median (IQR)	20.8 (4.38–81.2)	12.6 (2.40–64.0)	**<0.001**
Lymphocyte (cells/mm3), median (IQR)	79.2 (25.2–193)	75.7 (25.0–180)	0.196
Protein (mg/dL), median (IQR)	173 (105–314)	207 (124–350)	**0.001**
Glucose (mg/dL), median (IQR)	30.0 (19.0–47.2)	32.0 (22.0–45.0)	0.089

Continuous variables are displayed as median and interquartile ranges (IQR) whereas categorical variables are shown as absolute number and frequency (%). Data were compared between the clinical groups using the Mann–Whitney (continuous) or the Chi square (categorical) tests. TB, tuberculosis; CSF, cerebrospinal fluid; ARF, acute renal failure. Bold indicates *p* < 0.05.

Following lumbar puncture, both HIV- and HIV + groups exhibited predominantly clear CSF (56.5 and 63.7%, respectively - further characteristics of CSF appearance are shown in Supplementary Table S1). Upon laboratory analysis, the HIV− group displayed greater CSF leukocyte count (HIV−: 140 cells/mm^3^ [45–334]; HIV+: 93 [28–245], *p* < 0.001), neutrophil ratios (HIV−: 17%, IQR: 6–47; HIV+: 12, IQR: 3–35, *p* < 0.001), and neutrophil counts (HIV−: 21 cells/mm3, IQR:4–81; HIV+: 13, IQR: 2–64, *p* < 0.001). On the other hand, greater lymphocyte ratio values (HIV−: 76%, IQR: 38–90; HIV+: 82, IQR: 56–95, *p* < 0.001) and protein concentration (HIV−: 173 mg/dL, IQR: 105–315; HIV+: 207, IQR: 124–350, *p* = 0.001) were observed in the HIV + group. Differences in glucose concentration and lymphocyte count did not yield statistically significant results between groups.

### 3.2 Disease outcomes

The overall death rate of TBM for the population in this study was 19.8%. Death by meningitis occurred after a median of 7 days (IQR:0–20) from the notification date, whereas discharge took place after 15 days (IQR:3–28, *p* < 0.001). Among PLWH, the in-hospital mortality was 17.3%, whereas among HIV-negative individuals, 23.2% (*p* = 0.002). Deceased patients most likely displayed history of acute renal failure (ARF) (deceased: 3.58%; survived: 1.44%, *p* = 0.017), fever (deceased: 78.4%; survived: 71.8%, *p* = 0.016), seizures (deceased: 26.1%; survived: 14.3%, *p* < 0.001), nuchal rigidity (deceased: 40.6%; survived: 28.8%, *p* < 0.001), Kernig’s/Brudzinski’s sign (deceased: 8.95%; survived: 5.51%, *p* = 0.029) and coma (deceased: 23.9%; survived: 4.52%, *p* < 0.001), but less frequently reported headaches (deceased: 71.9%; survived: 79.7%, *p* = 0.003) when compared to patients who survived (Table 2).

**Table 2:** Characteristics of general population and comparison between deceased patients and survivors.

Characteristics	Deceased (*N* = 361)	Survived (*N* = 1,458)	*P* value
Age, median (IQR)	38.0 (31.0–49.0)	39.0 (31.0–48.0)	0.415
Sex (Male), *n* (%)	235 (65.1)	963 (66.0)	0.78
Race (Non-white), *n* (%)	158 (49.1)	669 (51.3)	0.52
Education (literate), *n* (%)	187 (95.4)	797 (96.3)	0.729
HIV, *n* (%)	179 (49.6)	855 (58.6)	**0.002**
ARF, *n* (%)	12 (3.58)	20 (1.44)	**0.017**
Previous TB, *n* (%)	155 (46.7)	669 (49.3)	0.428
Past Trauma, *n* (%)	10 (2.96)	25 (1.79)	0.25
Headache, *n* (%)	238 (71.9)	1,116 (79.7)	**0.003**
Fever, *n* (%)	269 (78.4)	1,011 (71.8)	**0.016**
Vomiting, *n* (%)	132 (39.9)	636 (45.7)	0.063
Seizures, *n* (%)	86 (26.1)	199 (14.3)	**<0.001**
Nuchal Rigidity, *n* (%)	134 (40.6)	398 (28.8)	**<0.001**
Kernig’s/Brudzinski’s sign, *n* (%)	29 (8.95)	75 (5.51)	**0.029**
Coma, *n* (%)	81 (23.9)	63 (4.52)	**<0.001**
Petechiae, *n* (%)	4 (1.19)	8 (0.57)	0.265
Symptom Onset, median (IQR)	11.0 (4.00–26.0)	13.0 (5.00–31.0)	0.114
Days to Outcome, median (IQR)	7.00 (0.00–20.2)	15.0 (3.00–28.0)	**<0.001**
CSF analysis			
Appearance (clear), *n* (%)	179 (53.8)	839 (62.3)	**0.041**
Leukocyte (cells/mm3), median (IQR)	110 (32.0–300)	110 (32.2–276)	0.719
Neutrophil (cells/mm3), median (IQR)	28.0 (5.22–100)	14.4 (2.81–65.2)	**<0.001**
Lymphocyte (cells/mm3), median (IQR)	67.2 (19.8–151)	79.9 (26.5–192)	**0.027**
Protein (mg/dL), median (IQR)	241 (138–444)	183 (111–312)	**<0.001**
Glucose (mg/dL), median (IQR)	27.0 (16.0–44.0)	32.0 (21.0–46.0)	**<0.001**

Continuous variables are displayed as median and interquartile ranges (IQR) whereas categorical variables are shown as absolute number and frequency (%). Data were compared between the clinical groups using the Mann–Whitney (continuous) or the Chi square (categorical) tests. TB, tuberculosis; CSF, cerebrospinal fluid; ARF, acute renal failure. Bold indicates *p* < 0.05.

Following CSF analysis, both groups (deceased vs. survived) displayed predominantly clear CSF (Supplementary Table S2). The deceased group exhibited greater neutrophil ratio (deceased: 23.0%, IQR:7–62; survived: 13.0, IQR:4–35, *p* < 0.001), neutrophil count (deceased: 28.0 cells/mm3, IQR:5–100; survived: 14, IQR:3–65, *p* < 0.001), and protein concentrations (deceased: 241 mg/dL, IQR:138–444; survived: 183, IQR:111–312, *p* < 0.001). Conversely, lymphocyte ratio (deceased: 68%, IQR: 31–90; survived: 81, IQR:55–93, *p* < 0.001), lymphocyte count (deceased: 67 cells/mm3, IQR:20–151; survived: 80, IQR:27–192, *p* = 0.027) and glucose concentration (deceased: 27 mg/dL, IQR:16–44; survived: 32 mg/dL, IQR:21–46, *p* < 0.001) displayed higher levels in those who survived. Differences in leukocyte count was not statistically significant between outcome groups.

Comparisons of disease outcomes within HIV-positive and HIV-negative populations demonstrated that the mortality rate for the HIV-negative population was 23.2%, differing from the HIV-positive population, whose death rate was 17.3%. With regards to clinical presentation, in both groups patients who survived were most likely to present with headache, while those who died were more likely to display coma (Supplementary Tables S3, S4). Furthermore, both populations also displayed similar CSF findings regarding disease outcomes, where those who died were prone to having higher neutrophil ratio, and counts (in the HIV-negative group, a borderline association was observed, with a *p*-value of 0.051) as well as greater protein concentrations. Similarly, greater lymphocyte ratios and glucose concentrations were observed in both HIV populations, albeit occurring predominantly in those who survived.

### 3.3 Risk factors associated with death by tuberculous meningitis

**Figure 2:**
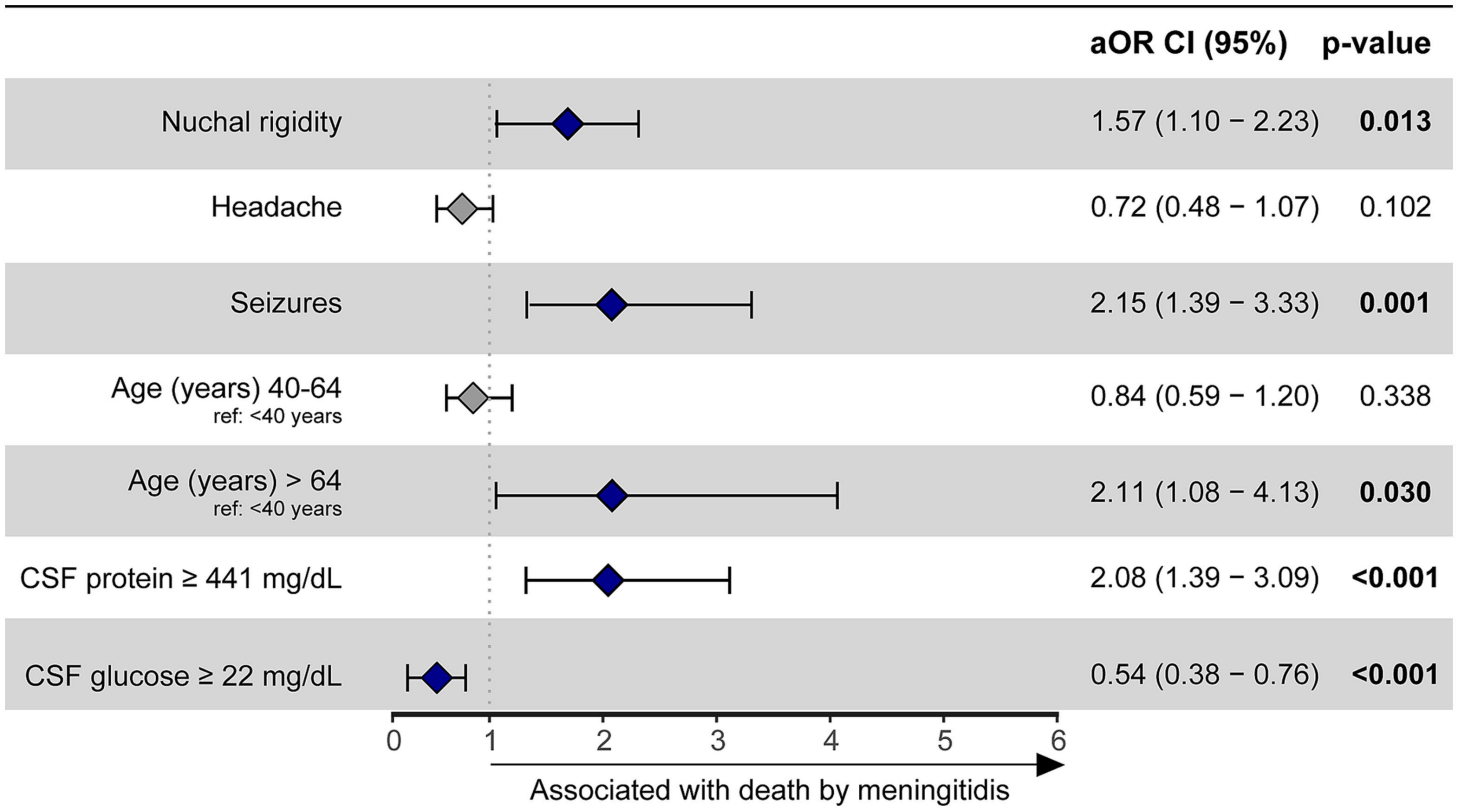
A binomial logistic regression with a stepwise method was used to evaluate which factors were associated with death by meningitis in the overall population (HIV positive and negative). Hospital discharge was considered as reference outcome. Seizures (reference: no seizures), nuchal rigidity (reference: no nuchal rigidity), age > 64 years (reference: age < 40 years) and CSF protein ≥ 441 mg/dL (reference: CSF protein < 441 mg/dL) were variables statistically associated with death by meningitis. CSF glucose ≥ 22 mg/dL (reference: CSF glucose <22 mg/dL) served as a protective factor against mortality. CSF, cerebrospinal fluid; aOR, adjusted odds ratio; CI, confidence interval.

To evaluate independent associations between sociodemographic, clinical and laboratory features with death by meningitis in a multivariate model, binomial logistic regression with a stepwise method analysis was employed. When adjusting for HIV status, our results concluded that no independent association was found between HIV and death by meningitis in this study population (Supplementary Table S5). Variables associated with death by meningitis included in the final model were: seizures (aOR: 2.15, 95%CI: 1.39–3.33, *p* < 0.001), nuchal rigidity (aOR: 1.57, 95%CI: 1.1–2.23, *p* = 0.014), age greater than 64 (aOR: 2.11, 95%CI: 1.08–4.13, *p* = 0.03) and CSF protein concentration ≥ 441 mg/dL (aOR: 2:08, 95%CI: 1.39–3.09, *p* < 0.001). On the other hand, a CSF glucose concentration ≥ 22 mg/dL was identified as a protective factor against mortality (aOR: 0.54, 95%CI: 0.38–0.76, *p* < 0.001) (Figure 2).

**Figure 3:**
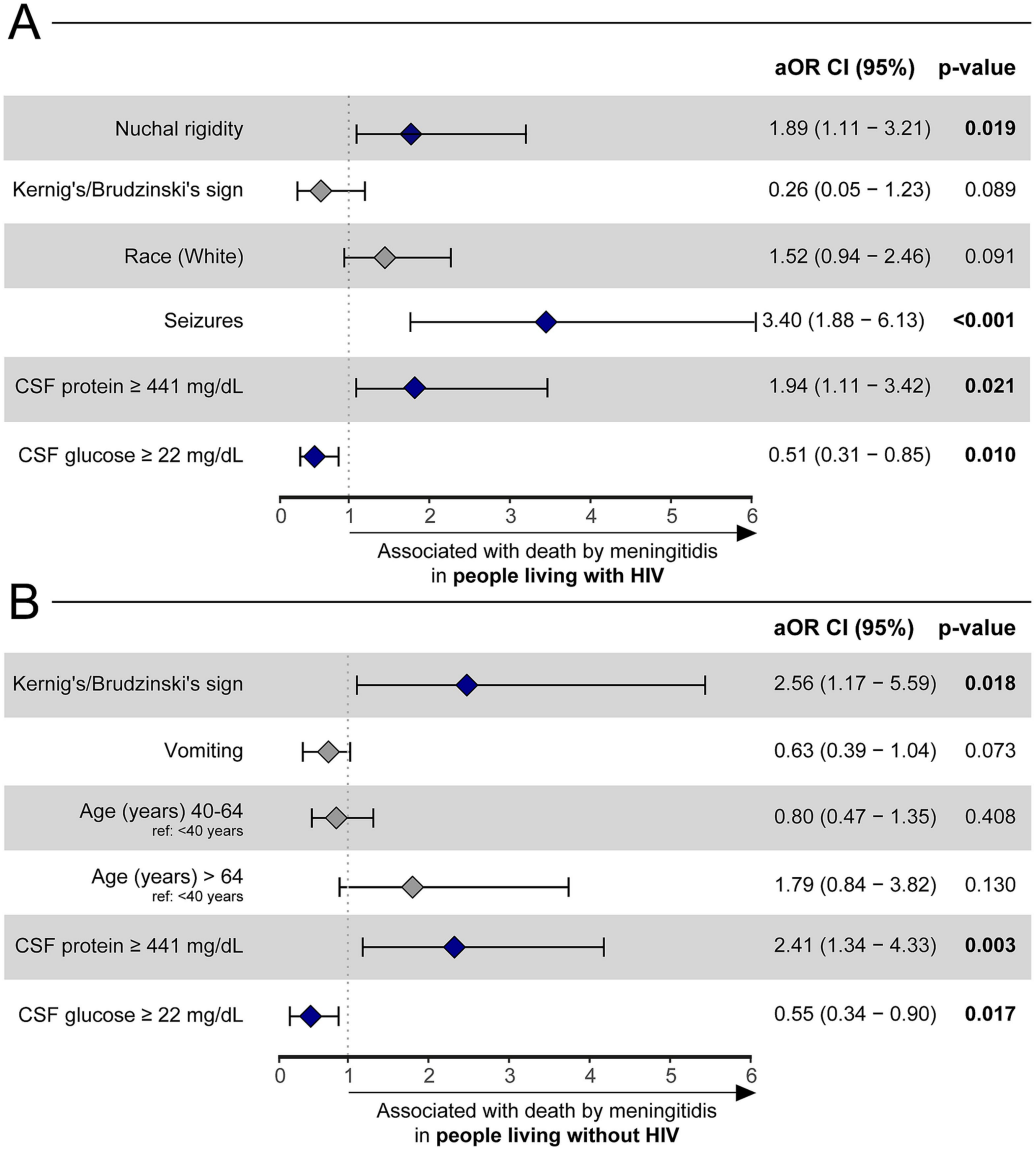
A binomial logistic regression with a stepwise method was used to evaluate which factors were associated with death by meningitidis among HIV-positive **(A)** and HIV-negative **(B)** adults with TBM. Only the final model of each analysis is shown. Hospital discharge was considered as reference outcome. Other references were: Seizures (reference: no seizures), nuchal rigidity (reference: no nuchal rigidity), CSF protein ≥ 441 mg/dL (reference: CSF protein < 441 mg/dL), CSF glucose ≥ 22 mg/dL (reference: CSF glucose <22 mg/dL), Kernig’s/Brudzinski’s sign (reference: no Kernig’s/Brudzinski’s sign), and age (reference: age < 40 years). HIV, human immunodeficiency virus. CSF, cerebrospinal fluid; aOR, adjusted odds ratio; CI, confidence interval.

Finally, two other logistic regressions were performed to assess independent variable associations with death by meningitis within each HIV-positive and HIV negative subpopulations (Supplementary Tables S6, S7). After stepwise selection, among the HIV-positive group, seizures (aOR: 3.4, 95%CI: 1.88, 6.13, *p* < 0.001), nuchal rigidity (aOR: 1.89, 95%CI: 1.11, 3.21) and CSF protein concentration ≥441 mg/dL (aOR: 1.94, 95%CI: 1.11–3.42, *p* = 0.024) were associated with death by meningitis, whereas a CSF glucose concentration ≥22 mg/dL (aOR: 0.51, 95%CI: 0.31–0.85, *p* = 0.01) served as beneficial against mortality (Figure 3A). Logistic regression in the HIV-negative group demonstrated that Kernig’s/Brudzinski’s sign (aOR: 2.56, 95%CI: 1.17–5.59, *p* = 0.023) and CSF protein concentration ≥441 mg/dL (aOR: 2.41, 95%CI: 1.34–4.33, *p* = 0.004) were related to death by meningitis. Similar to the HIV-positive group, a CSF glucose concentration ≥22 mg/dL was also an indicator of improved outcomes (aOR: 0.55, 95%CI: 0.34–0.9, *p* = 0.017) within HIV-negative individuals (Figure 3B).

## 4 Discussion

In this study, we investigated the epidemiologic and clinical characteristics of Brazilian patients diagnosed with TBM using data from the SINAN-meningitis database (2007–2021). Specifically, we explored differences in clinical and laboratory presentations between HIV-positive and HIV-negative patients and identified factors associated with mortality. Although various studies have examined TBM considering HIV status, to our knowledge, none have employed a nationwide approach in Brazil, which could provide valuable insights for directing clinical and administrative efforts. Our findings revealed a high frequency of HIV co-infection (56.8%) among adult TBM cases in Brazil. This aligns with expectations given Brazil’s position as one of the 30 countries with the highest TB/HIV burden (12) and the increased risk for developing TBM in TB/HIV coinfected individuals (5). Similar co-infection frequencies have been reported in other low- and middle-income countries, ranging from 38 to 55.7% (9, 10, 18, 19). Additionally, in our study, PLWH were younger and more likely to have a previous diagnosis of TB, consistent with previous findings (9, 10, 19).

Compared to HIV-negative patients, PLWH displayed a distinct clinical profile, with a lower likelihood of manifesting classic signs of TBM, such as vomiting, nuchal rigidity, meningeal inflammation, and coma. This contradicts some previous studies (9, 10) but may be explained by the immune dysregulation caused by HIV, given that the clinical manifestations of TBM arise from the host immune response to MTB in the CNS. Specifically, the host immune response results in granulomatous inflammation in the basal meninges. Inflammatory exudates may obstruct CSF absorption by subarachnoid cisterns and lead to hydrocephalus (20), a common manifestation of which is vomiting and headache due to increased intracranial pressure. Given that PLWH has been shown to have impaired granuloma formation (21), a decreased occurrence of vomiting and a trend for decreased headaches, is plausible. This diminished immune response may also explain the reduced occurrence of nuchal rigidity, which is reflective of subarachnoid inflammation. Further studies are warranted to further investigate the pathogenesis of such findings.

In line with these findings, PLWH also exhibited a distinct CSF profile, particularly a lower total leukocyte count. This has been previously noted, with some studies suggesting that HIV modulates both the quantitative and qualitative aspects of the intracerebral immune response (22). Although the precise mechanism remains unclear, our results underscore the need for heightened clinical suspicion in PLWH, given their atypical presentations and more subtle elevations in CSF leukocyte count. Diagnosing TBM in this population often relies on subtle clinical and CSF findings, making it crucial to detect these patterns early.

Regarding mortality, our univariate analysis identified fever and advanced disease stages (e.g., seizures, nuchal rigidity, coma) as associated with higher mortality risk, consistent with previous studies linking advanced TBM stages (BMRC II/III) (23) to poorer outcomes (19, 24). Multivariate analysis confirmed that advanced age, seizures, and nuchal rigidity were independent predictors of death, corroborating other studies that have linked these factors to higher mortality (4, 25). These results emphasize the importance of early diagnosis and treatment, particularly in older adult patients and those with signs of advanced disease.

Interestingly, while fever was associated with mortality in the univariate analysis, this relationship did not persist in the multivariate model. Although some studies have suggested a link between fever and mortality (26), the role of fever in TBM prognosis remains unclear, and further research is warranted to evaluate the potential benefits of antipyretic therapy in these cases.

Laboratory parameters also played a key role in predicting mortality. High CSF protein levels and low CSF glucose concentrations were both independent predictors of death, regardless of HIV status. Elevated CSF protein is a hallmark of meningitis and reflects blood–brain barrier dysfunction and meningeal inflammation (27). This association has been documented in previous studies (28), highlighting the importance of protein elevation as a marker of advanced disease (22). Similarly, hypoglycorrhachia, indicative of increased glucose consumption, impaired transport across the blood–brain barrier and cell metabolism shifts from inflammation-induced damage and ischemia (29), was also associated with increased mortality.

Our study revealed an overall mortality rate of 19.8%, consistent with similar rates reported in the literature (25). However, we found that PLWH had a lower in-hospital mortality rate (17.3%) compared to HIV-negative patients (23.2%), and HIV co-infection was not an independent predictor of mortality. This contrasts with findings from other studies where HIV status was linked to higher mortality (4, 5). The discrepancy may be due to differences in study design, as we focused on in-hospital mortality, while other studies examined longer-term outcomes (e.g., 6-month or 1-year mortality) (5). Additionally, Brazil’s substantial progress in HIV care over the past decade may have contributed to a less symptomatic presentation and the lower mortality rate observed in our study. Antiretroviral therapy (ART) has been available and cost-free in the country since 1996, and starting in 2013, became accessible to all PLWH regardless of CD4 count (30). According to the Brazilian Ministry of Health’s 2022 HIV Clinical Monitoring Report (31), ART coverage among PLWH increased from 65.6% in 2013 to 81.4% in 2022. In addition, among those on ART within the last 6 months, 95.1% achieved viral suppression (i.e., viral load <1,000 copies/mL) in 2022, compared to 85.4% in 2013. During this period, the median time between the first measured CD4 count, and ART initiation plummeted from 322 days in 2012 to just 21 days in 2022. Moreover, since 2014, over two-thirds of PLWH initiated ART with CD4 counts above 500 cells/mm^3^. Hence, these improvements suggest that many PLWH are being diagnosed and treated earlier, maintaining better immune function. Such characteristics may contribute to a reduction in AIDS-related mortality, including TBM. Therefore, the improved survival among PLWH in our cohort likely reflect the cumulative impact of expanded ART coverage, better immune function, earlier treatment initiation, and potentially closer medical monitoring during hospitalization.

Despite the valuable insights provided by this study, several limitations must be acknowledged. Missing data on key demographic and clinical variables, such as substance use, homelessness, and detailed neurological assessments, limited the comprehensiveness of our analyses. Missing data for CSF parameters also reduced our sample size. Furthermore, the lack of imaging data and detailed clinical assessments (e.g., cranial nerve palsies, Glasgow coma scale scores) prevented us from categorizing cases using the uniform TBM case definition (32). Other limitation is that TBM cases could have been notified in the SINAN-TB database instead of SINAN-meningitidis, thus resulting in significant TBM underreporting. The SINAN-TB database allows for the notification of both pulmonary and extrapulmonary TB. Some patients with both pulmonary and TBM may have been reported solely as having pulmonary TB, and further - not be additionally notified in the SINAN-meningitis database. Additionally, information on TBM treatment regimen, ART adherence, CD4 counts, and viral loads, as well as corticosteroid use and the presence of drug-resistant TB strains, were unavailable, potentially influencing our results. The SINAN-meningitis form includes only whether HIV infection was present at the time of TBM notification, without distinguishing between newly diagnosed cases and individuals already known to have the diagnosis. More specific aspects on HIV diagnosis and ART usage are available in other SINAN databases (SINAN-HIV and SINAN-AIDS), however, the anonymous nature of these datasets hinders the ability to track patients across datasets at the individual level. As a result, we were unable to assess for ART coverage, immune status, or healthcare follow-up for PLWH in our cohort. While we speculate that widespread ART availability, based on ART coverage epidemiological studies in the country, may have contributed to the observed outcomes in our study, this cannot be verified through our dataset. Therefore, our findings may not be generalizable to other HIV-associated TBM populations and should be interpreted with caution, particularly when comparing to cohorts in other countries with high-prevalence settings and low ART coverage rates. Finally, out-of-hospital mortality was not captured, possibly underestimating overall mortality rates due to post-TBM complications and the undiagnosed presence or development of opportunistic infections after discharge. This reflects another limitation of the SINAN databases, which do not allow for individual-level follow-up tracking and thus do not capture outcomes beyond the initial hospitalization.

In conclusion, our study indicates that while TBM is more prevalent among Brazilian PLWH, they tend to exhibit fewer classical symptoms and have lower in-hospital mortality. HIV was not an independent predictor of mortality in this population, but factors such as seizures, nuchal rigidity, advanced age, and elevated CSF protein were associated with higher mortality. These findings underscore the importance of maintaining a high level of clinical suspicion in PLWH and of identifying key risk factors early to improve TBM outcomes in Brazil. However, given the limitations of our study, particularly regarding ART coverage and immune status, the generalizability of these findings to other settings remains limited. Notably, the results observed may be influenced by Brazil’s substantial scale-up of HIV care, including the nationwide provision of free ART to all PLWH, high treatment coverage, and high rates of viral suppression. Further studies in cohorts with detailed data on ART use and immunological status in regions with well-established high ART coverage are needed to improve our understanding of TBM in PLWH.

## Data Availability

The datasets presented in this study can be found in online repositories. The names of the repository/repositories and accession number(s) can be found below: The datasets analyzed for this study are part of Brazil’s national meningitidis surveillance system, of which data is publicly available and can be found in: https://datasus.saude.gov.br/transferencia-de-arquivos/.
