# Supplementary material for: Influence of HIV co-infection on clinical presentation and disease outcome in hospitalized adults with tuberculous meningitis in Brazil: a nationwide observational study
Source: Front Public Health. 2025 Jun 16;13:1600104. doi: 10.3389/fpubh.2025.1600104 (PMC12206807; doi:10.3389/fpubh.2025.1600104)
Supplement: Supplementary file 1 [file Supplementary_file_1.DOCX]

Supplementary Material

**Supplementary figure 1:** Study flowchart.


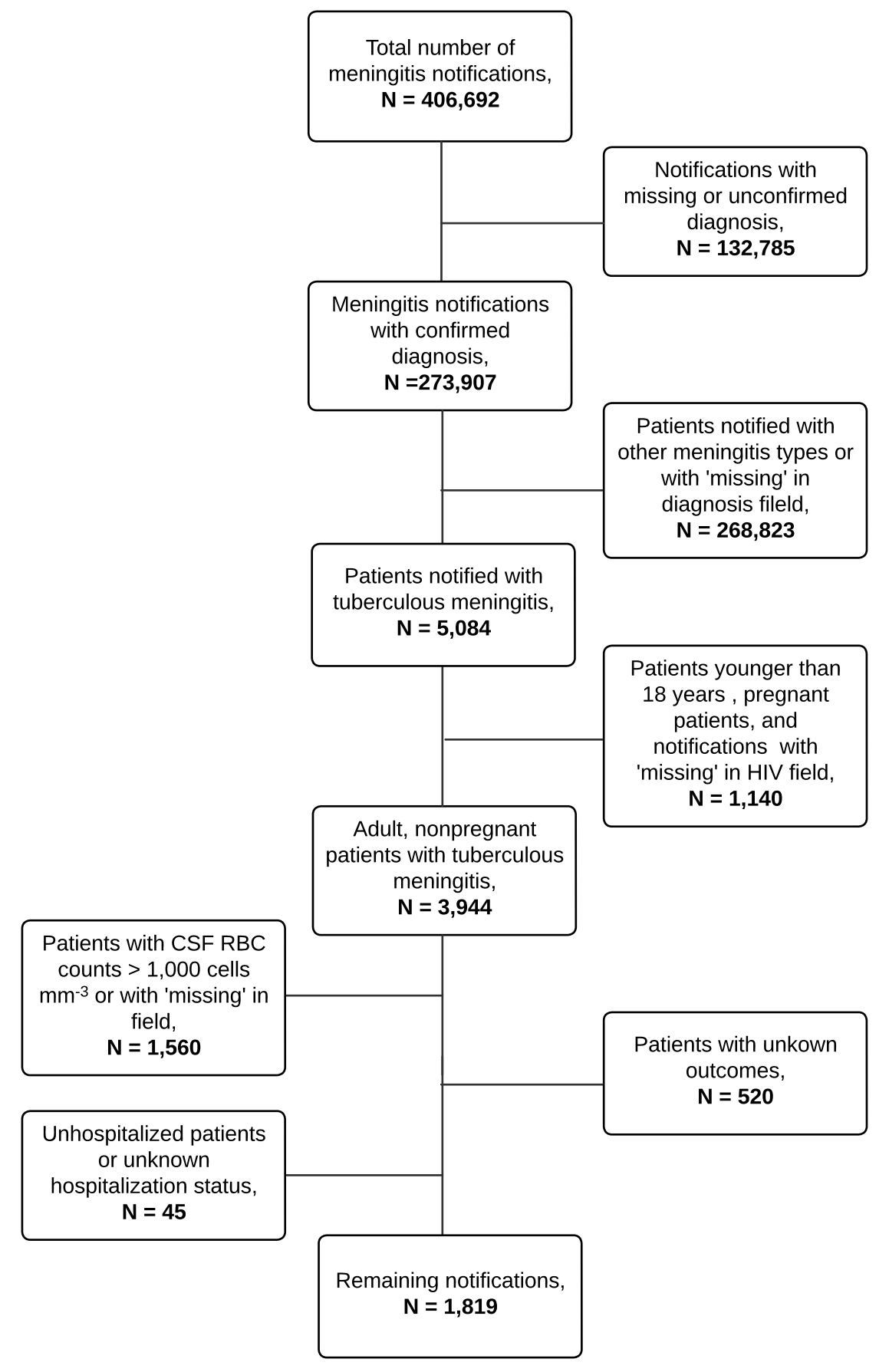


Abbreviations: HIV: human immunodeficiency virus. CSF: cerebrospinal fluid. RBC: red blood cell.

**Supplementary table 1:** Comparison of CSF appearance between HIV-positive and HIV-negative adults with TBM.

| **Characteristics** | **HIV negative (N=785)** | **HIV positive (N=1034)** | **P value** |
| --- | --- | --- | --- |
|  |  |  |  |
| **CSF Appearance, n (%):** |  | | **0.001** |
| **Bloody** | 10 (1.39) | 8 (0.83) |  |
| **Clear** | 406 (56.5) | 612 (63.7) |  |
| **Cloudy** | 212 (29.5) | 237 (24.7) |  |
| **Other** | 33 (4.59) | 27 (2.81) |  |
| **Purulent** | 1 (0.14) | 3 (0.31) |  |
| **Yellow** | 57 (7.93) | 74 (7.70) |  |

Table note: Categorical variables are shown as absolute number and frequency (%) and were compared between the clinical groups using the the Chi square (categorical) test. Abbreviations: CSF: cerebrospinal fluid.

**Supplementary table 2:** Comparison of CSF appearance between deceased patients and survivors.

| **Characteristics** | **Deceased (N=361)** | **Survived (N=1458)** | **P value** |
| --- | --- | --- | --- |
|  |  |  |  |
| **CSF Appearance, n (%):** |  | | **0.041** |
| **Bloody** | 3 (0.90) | 15 (1.11) |  |
| **Clear** | 179 (53.8) | 839 (62.3) |  |
| **Cloudy** | 100 (30.0) | 349 (25.9) |  |
| **Other** | 16 (4.80) | 44 (3.27) |  |
| **Purulent** | 0 (0.00) | 4 (0.30) |  |
| **Yellow** | 35 (10.5) | 96 (7.13) |  |

Table note: Categorical variables are shown as absolute number and frequency (%) and were compared between the clinical groups using the the Chi square (categorical) test. Abbreviations: CSF: cerebrospinal fluid.

**Supplementary table 3:** Comparison between characteristics of deceased patients and survivors within the PWH group.

| **Characteristics** | **Deceased (N=179)** | **Survived (N=855)** | **P value** |
| --- | --- | --- | --- |
|  |  |  |  |
| **Age, median (IQR)** | 37.0 (32.5-45.0) | 38.0 (32.0-45.0) | 0.999 |
| **Sex (Male), n (%)** | 112 (62.6) | 584 (68.3) | 0.162 |
| **Race (Non-white), n (%)** | 80 (48.8) | 408 (52.2) | 0.471 |
| **Education (literate), n (%)** | 101 (93.5) | 489 (96.3) | 0.193 |
| **ARF, n (%)** | 3 (1.88) | 11 (1.36) | 0.714 |
| **Previous TB, n (%)** | 87 (55.1) | 468 (58.9) | 0.425 |
| **Past Trauma, n (%)** | 4 (2.45) | 12 (1.48) | 0.325 |
| **Headache, n (%)** | 114 (69.5) | 639 (77.9) | **0.026** |
| **Fever, n (%)** | 137 (80.1) | 585 (71.3) | **0.024** |
| **Vomiting, n (%)** | 69 (42.1) | 335 (41.1) | 0.886 |
| **Seizures, n (%)** | 50 (31.2) | 110 (13.5) | **<0.001** |
| **Nuchal Rigidity, n (%)** | 62 (38.0) | 191 (23.7) | **<0.001** |
| **Kernig's/Brudzinski's sign, n (%)** | 7 (4.32) | 39 (4.88) | 0.921 |
| **Coma, n (%)** | 35 (20.8) | 29 (3.55) | **<0.001** |
| **Petechiae, n (%)** | 2 (1.20) | 4 (0.49) | 0.268 |
| **Symptom Onset, median (IQR)** | 9.00 (3.00-22.5) | 14.0 (5.00-33.0) | **0.004** |
| **Days to Outcome, median (IQR)** | 6.00 (1.00-18.8) | 14.0 (3.00-27.0) | **<0.001** |
| **CSF Analysis** | | | |
| **Appearance (clear), n (%)** | 89 (54.9) | 523 (65.5) | **0.049** |
| **Leukocyte (cells/mm3), median (IQR)** | 101 (32.5-298) | 93.0 (27.0-236) | **0.205** |
| **Neutrophil (cells/mm3), median (IQR)** | 22.8 (5.14-95.1) | 11.2 (2.10-56.0) | **0.004** |
| **Lymphocyte (cells/mm3), median (IQR)** | 79.6 (29.8-169) | 75.0 (23.0-181) | 0.615 |
| **Protein (mg/dL), median (IQR)** | 259 (149-464) | 198 (118-332) | **0.001** |
| **Glucose (mg/dL), median (IQR)** | 29.0 (18.0-44.0) | 33.0 (23.0-45.0) | **0.019** |

Table note: Continuous variables are displayed as median and interquartile ranges (IQR) whereas categorical variables are shown as absolute number and frequency (%). Data were compared between the clinical groups using the Mann-Whitney (continuous) or the Chi square (categorical) tests. Abbreviations: TB: tuberculosis. CSF: cerebrospinal fluid.

**Supplementary table 4:** Comparison between characteristics of deceased patients and survivors within the HIV-negative group.

| **Characteristics** | **Deceased (N=182)** | **Survived (N=603)** | **P value** |
| --- | --- | --- | --- |
|  |  |  |  |
| **Age, median (IQR)** | 40.0 (28.2-56.0) | 39.0 (29.0-53.0) | 0.427 |
| **Sex (Male), n (%)** | 123 (67.6) | 379 (62.9) | 0.282 |
| **Race (Non-white), n (%)** | 78 (49.4) | 261 (49.8) | 0.995 |
| **Education (literate), n (%)** | 86 (97.7) | 308 (96.2) | 0.743 |
| **ARF, n (%)** | 9 (5.14) | 9 (1.55) | **0.019** |
| **Previous TB, n (%)** | 68 (39.1) | 201 (35.8) | 0.482 |
| **Past Trauma, n (%)** | 6 (3.43) | 13 (2.23) | 0.408 |
| **Headache, n (%)** | 124 (74.3) | 477 (82.1) | **0.032** |
| **Fever, n (%)** | 132 (76.7) | 426 (72.4) | 0.306 |
| **Vomiting, n (%)** | 63 (37.7) | 301 (52.3) | **0.001** |
| **Seizures, n (%)** | 36 (21.2) | 89 (15.5) | 0.101 |
| **Nuchal Rigidity, n (%)** | 72 (43.1) | 207 (35.9) | 0.107 |
| **Kernig's/Brudzinski's sign, n (%)** | 22 (13.6) | 36 (6.43) | **0.005** |
| **Coma, n (%)** | 46 (26.9) | 34 (5.90) | **<0.001** |
| **Petechiae, n (%)** | 2 (1.17) | 4 (0.69) | 0.625 |
| **Symptom Onset, median (IQR)** | 13.0 (6.00-26.0) | 12.0 (6.00-28.0) | 0.395 |
| **Days to Outcome, median (IQR)** | 8.00 (0.00-21.0) | 15.0 (5.00-28.0) | **<0.001** |
| **CSF Analysis** | | | |
| **Appearance (clear), n (%)** | 90 (52.6) | 316 (57.7) | 0.415 |
| **Leukocyte (cells/mm3), median (IQR)** | 122 (28.2-295) | 144 (52.0-340) | **0.156** |
| **Neutrophil (cells/mm3), median (IQR)** | 29.7 (5.94-104) | 18.8 (3.96-75.0) | 0.051 |
| **Lymphocyte (cells/mm3), median (IQR)** | 49.1 (14.3-136) | 87.0 (30.3-217) | **<0.001** |
| **Protein (mg/dL) , median (IQR)** | 211 (124-435) | 163 (102-296) | **<0.001** |
| **Glucose (mg/dL) , median (IQR)** | 25.0 (15.0-42.0) | 31.0 (20.0-49.0) | **0.015** |

Table note: Continuous variables are displayed as median and interquartile ranges (IQR) whereas categorical variables are shown as absolute number and frequency (%). Data were compared between the clinical groups using the Mann-Whitney (continuous) or the Chi square (categorical) tests. Abbreviations: TB: tuberculosis. CSF: cerebrospinal fluid.

**Supplementary table 5:** Table including the initial regression model for the general population.

| Variable | adj.OR | (95%CI) | P(Wald's test) | P(LR-test) |
| --- | --- | --- | --- | --- |
|  |  |  |  |  |
| Sex (Male) | 0.93 | (0.65-1.34) | 0.706 | 0.707 |
| Race (White) | 1.27 | (0.91-1.79) | 0.164 | 0.163 |
| HIV | 0.85 | (0.59-1.21) | 0.358 | 0.358 |
| Previous TB | 0.98 | (0.69-1.4) | 0.921 | 0.921 |
| Headache | 0.71 | (0.47-1.09) | 0.114 | 0.117 |
| Fever | 1.33 | (0.88-1.99) | 0.174 | 0.168 |
| Vomiting | 0.88 | (0.61-1.26) | 0.492 | 0.492 |
| Seizures | 2.2 | (1.41-3.42) | < 0.001 | < 0.001 |
| Nuchal Rigidity | 1.46 | (1-2.15) | 0.051 | 0.052 |
| Kernig's/Brudzinski's sign | 1.24 | (0.62-2.45) | 0.543 | 0.548 |
| CSF Glucose ≥22 (reference: <22 mg/dL) | 0.53 | (0.37-0.76) | < 0.001 | < 0.001 |
| Age (reference: ≤39) |  |  |  | 0.074 |
| >64 | 1.87 | (0.93-3.77) | 0.081 |  |
| 40-64 | 0.82 | (0.58-1.18) | 0.29 |  |
| CSF Leukocytes (reference: ≤36 cells/mm3) |  |  |  | 0.948 |
| >64 | 0.94 | (0.59-1.51) | 0.812 |  |
| 36-64 | 1.02 | (0.53-1.95) | 0.957 |  |
| CSF Protein ≥441 (reference: <441 mg/dL) | 2.19 | (1.46-3.28) | < 0.001 | < 0.001 |

Abbreviations: adj.OR: adjusted odds ratio. CI: confidence interval. TB: tuberculosis. CSF: cerebrospinal fluid. LR: likelihood ratio.

**Supplementary table 6:** Table including the initial regression model within the HIV-positive population.

| **Variable:** | **adj.OR** | **(95%CI)** | **P(Wald's test)** | **P(LR-test)** |
| --- | --- | --- | --- | --- |
|  |  |  |  |  |
| **Sex (Male)** | 0.75 | (0.45-1.25) | 0.27 | 0.272 |
| **Race (White)** | 1.51 | (0.93-2.47) | 0.096 | 0.095 |
| **Previous TB** | 0.86 | (0.52-1.42) | 0.554 | 0.553 |
| **Headache** | 0.75 | (0.41-1.37) | 0.347 | 0.351 |
| **Fever** | 1.48 | (0.81-2.69) | 0.201 | 0.192 |
| **Vomiting** | 0.96 | (0.57-1.61) | 0.883 | 0.883 |
| **Seizures** | 3.47 | (1.87-6.43) | < 0.001 | < 0.001 |
| **Nuchal Rigidity** | 1.77 | (1.01-3.08) | 0.045 | 0.048 |
| **Kernig's/Brudzinski's sign** | 0.28 | (0.06-1.36) | 0.113 | 0.074 |
| **CSF Glucose ≥22 (reference: <22 mg/dL)** | 0.51 | (0.3-0.86) | 0.012 | 0.013 |
| **Age (reference: ≤39)** |  |  |  | 0.71 |
| **>64** | 1.14 | (0.11-11.45) | 0.911 |  |
| **40-64** | 0.81 | (0.5-1.34) | 0.42 |  |
| **CSF Leukocytes (reference: ≤36 cells/mm3)** |  |  |  | 0.509 |
| **>64** | 1.39 | (0.69-2.78) | 0.356 |  |
| **36-64** | 1.66 | (0.67-4.11) | 0.269 |  |
| **CSF Protein ≥441 (reference: <441 mg/dL)** | 1.99 | (1.11-3.54) | 0.02 | 0.023 |

Abbreviations: adj.OR: adjusted odds ratio. CI: confidence interval. TB: tuberculosis. CSF: cerebrospinal fluid. LR: likelihood ratio.

**Supplementary table 7:** Table including the initial regression model of the HIV-negative population.

| **Variable:** | **adj.OR** | **(95%CI)** | **P(Wald's test)** | **P(LR-test)** |
| --- | --- | --- | --- | --- |
|  |  |  |  |  |
| **Sex (Male)** | 1.11 | (0.66-1.88) | 0.696 | 0.695 |
| **Race (White)** | 1.00 | (0.6121-1.6512) | 0.983 | 0.983 |
| **Previous TB** | 1.1 | (0.65-1.85) | 0.724 | 0.724 |
| **Headache** | 0.73 | (0.39-1.36) | 0.318 | 0.322 |
| **Fever** | 1.13 | (0.63-2.02) | 0.679 | 0.678 |
| **Vomiting** | 0.69 | (0.41-1.16) | 0.159 | 0.158 |
| **Seizures** | 1.46 | (0.75-2.86) | 0.265 | 0.273 |
| **Nuchal Rigidity** | 1.27 | (0.74-2.19) | 0.379 | 0.38 |
| **Kernig's/Brudzinski's sign** | 2.63 | (1.14-6.03) | 0.023 | 0.027 |
| **CSF Glucose ≥22 (reference: <22 mg/dL)** | 0.55 | (0.34-0.91) | 0.021 | 0.021 |
| **Age (reference: ≤39)** |  |  |  | 0.178 |
| **>64** | 1.65 | (0.76-3.61) | 0.209 |  |
| **40-64** | 0.79 | (0.46-1.35) | 0.386 |  |
| **CSF Leukocytes (reference: ≤36 cells/mm3)** |  |  |  | 0.339 |
| **>64** | 0.61 | (0.31-1.2) | 0.15 |  |
| **36-64** | 0.57 | (0.21-1.55) | 0.273 |  |
| **CSF Protein ≥441 (reference: <441 mg/dL)** | 2.53 | (1.39-4.61) | 0.002 | 0.003 |

Abbreviations: adj.OR: adjusted odds ratio. CI: confidence interval. TB: tuberculosis. CSF: cerebrospinal fluid. LR: likelihood ratio.

**Supplementary table 8:** Table including crude and adjusted odds ratio (OR) from the final regression model for the general population.

| **Variable:** | **Crude OR** | **(95% CI)** | **adj.OR** | **(95%CI)** | **P(Wald's test)** | **P(LR-test)** |
| --- | --- | --- | --- | --- | --- | --- |
| Headache | 0.69 | (0.47-1) | 0.72 | (0.48-1.07) | 0.102 | 0.106 |
| Seizures | 2.31 | (1.52-3.52) | 2.15 | (1.39-3.33) | < 0.001 | < 0.001 |
| Nuchal Rigidity | 1.4 | (1-1.96) | 1.57 | (1.1-2.23) | 0.013 | 0.014 |
| CSF glucose ≥ 22 mg/dL | 0.5 | (0.36-0.7) | 0.54 | (0.38-0.76) | < 0.001 | < 0.001 |
| Age (reference: ≤39) |  | | | | | 0.035 |
| >64 | 2.36 | (1.24-4.47) | 2.11 | (1.08-4.13) | 0.03 | - |
| 40-64 | 0.89 | (0.63-1.25) | 0.84 | (0.59-1.2) | 0.338 | - |
| CSF Protein ≥441 (reference: <441 mg/dL) | 2.26 | (1.54-3.31) | 2.08 | (1.39-3.09) | < 0.001 | < 0.001 |

Abbreviations: OR: odds ratio. adj.OR: adjusted odds ratio. CI: confidence interval. CSF: cerebrospinal fluid. LR: likelihood ratio.

**Supplementary table 9:** Table including crude and adjusted odds ratio (OR) from the final regression model of the HIV-negative population.

| **Variable:** | **Crude OR** | **(95% CI)** | **adj.OR** | **(95%CI)** | **P(Wald's test)** | **P(LR-test)** |
| --- | --- | --- | --- | --- | --- | --- |
| **Vomiting** | 0.61 | (0.38-0.97) | 0.63 | (0.39-1.04) | 0.073 | 0.071 |
| **Kernig's/Brudzinski's sign** | 1.88 | (0.9-3.94) | 2.56 | (1.17-5.59) | 0.018 | 0.023 |
| **CSF Glucose ≥22 (reference: <22 mg/dL)** | 0.53 | (0.33-0.84) | 0.55 | (0.34-0.9) | 0.017 | 0.017 |
| **Age (reference: ≤39)** |  |  |  |  |  | 0.124 |
| **>64** | 2.32 | (1.14-4.71) | 1.79 | (0.84-3.82) | 0.13 |  |
| **40-64** | 0.92 | (0.56-1.52) | 0.8 | (0.47-1.35) | 0.408 |  |
| **CSF Protein ≥441 (reference: <441 mg/dL)** | 2.58 | (1.48-4.51) | 2.41 | (1.34-4.33) | 0.003 | 0.004 |

Abbreviations: OR: odds ratio. adj.OR: adjusted odds ratio. CI: confidence interval. CSF: cerebrospinal fluid. LR: likelihood ratio.

**Supplementary table 10:** Table including crude and adjusted odds ratio (OR) from the final regression model of the HIV-positive population.

| **Variable:** | **Crude OR** | **(95% CI)** | **adj.OR** | **(95%CI)** | **P(Wald's test)** | **P(LR-test)** |
| --- | --- | --- | --- | --- | --- | --- |
| **Race (White)** | 1.43 | (0.9-2.27) | 1.52 | (0.94-2.46) | 0.091 | 0.089 |
| **Seizures** | 3.37 | (1.91-5.95) | 3.4 | (1.88-6.13) | < 0.001 | < 0.001 |
| **Nuchal Rigidity** | 1.48 | (0.91-2.42) | 1.89 | (1.11-3.21) | 0.019 | 0.021 |
| **Kernig's/Brudzinski's sign** | 0.45 | (0.1-1.98) | 0.26 | (0.05-1.23) | 0.089 | 0.052 |
| **CSF Glucose ≥22 (reference: <22 mg/dL)** | 0.5 | (0.31-0.82) | 0.51 | (0.31-0.85) | 0.01 | 0.012 |
| **CSF Protein ≥441 (reference: <441 mg/dL)** | 2.07 | (1.21-3.52) | 1.94 | (1.11-3.42) | 0.021 | 0.024 |

Abbreviations: OR: odds ratio. adj.OR: adjusted odds ratio. CI: confidence interval. CSF: cerebrospinal fluid. LR: likelihood ratio.
